# Science Objectives for Flagship-Class Mission Concepts for the Search for Evidence of Life at Enceladus

**DOI:** 10.1089/ast.2020.2425

**Published:** 2022-06-08

**Authors:** Shannon M. MacKenzie, Marc Neveu, Alfonso F. Davila, Jonathan I. Lunine, Morgan L. Cable, Charity M. Phillips-Lander, Jennifer L. Eigenbrode, J. Hunter Waite, Kate L. Craft, Jason D. Hofgartner, Chris P. McKay, Christopher R. Glein, Dana Burton, Samuel P. Kounaves, Richard A. Mathies, Steven D. Vance, Michael J. Malaska, Robert Gold, Christopher R. German, Krista M. Soderlund, Peter Willis, Caroline Freissinet, Alfred S. McEwen, John Robert Brucato, Jean-Pierre P. de Vera, Tori M. Hoehler, Jennifer Heldmann

**Affiliations:** ^1^ Johns Hopkins University Applied Physics Laboratory, Laurel, Maryland, USA.; ^2^ Department of Astronomy, University of Maryland, College Park, Maryland, USA.; ^3^ Solar System Exploration Division, NASA Goddard Space Flight Center, Greenbelt, Maryland, USA.; ^4^ Division of Space Science and Astrobiology, NASA Ames Research Center, Moffett Field, California, USA.; ^5^ Department of Astronomy, Cornell University, Ithaca, New York, USA.; ^6^ Carl Sagan Institute, Cornell University, Ithaca, New York, USA.; ^7^ Jet Propulsion Laboratory, California Institute of Technology, Pasadena, California, USA.; ^8^ Space Science and Engineering Division, Southwest Research Institute, San Antonio, Texas, USA.; ^9^ Department of Anthropology, George Washington University, Washington, District of Columbia, USA.; ^10^ Department of Chemistry, Tufts University, Medford, Massachusetts, USA.; ^11^ Chemistry Department and Space Sciences Laboratory, University of California, Berkeley, Berkeley, California, USA.; ^12^ Department of Geology & Geophysics, Woods Hole Oceanographic Institution, Woods Hole, Massachusetts, USA.; ^13^ Institute for Geophysics, Jackson School of Geosciences, The University of Texas at Austin, Austin, Texas, USA.; ^14^ CNRS-University of Versailles St Quentin, LATMOS, Guyancourt, France.; ^15^ Lunar and Planetary Lab, University of Arizona, Tucson, Arizona, USA.; ^16^ INAF-Astrophysical Observatory of Arcetri, Firenze, Italy.; ^17^ Space Operations and Astronaut Training, MUSC, German Aerospace Center (DLR), Cologne, Germany.

**Keywords:** Enceladus, Mission, Life detection, Habitability

## Abstract

*Cassini* revealed that Saturn's Moon Enceladus hosts a subsurface ocean that meets the accepted criteria for habitability with bio-essential elements and compounds, liquid water, and energy sources available in the environment. Whether these conditions are sufficiently abundant and collocated to support life remains unknown and cannot be determined from *Cassini* data. However, thanks to the plume of oceanic material emanating from Enceladus’ south pole, a new mission to Enceladus could search for evidence of life without having to descend through kilometers of ice. In this article, we outline the science motivations for such a successor to *Cassini*, choosing the primary science goal to be determining whether Enceladus is inhabited and assuming a resource level equivalent to NASA's Flagship-class missions. We selected a set of potential biosignature measurements that are complementary and orthogonal to build a robust case for any life detection result. This result would be further informed by quantifications of the habitability of the environment through geochemical and geophysical investigations into the ocean and ice shell crust. This study demonstrates that Enceladus’ plume offers an unparalleled opportunity for *in situ* exploration of an Ocean World and that the planetary science and astrobiology community is well equipped to take full advantage of it in the coming decades.

## 1. Introduction

The search for evidence of life elsewhere in the Solar System as a concerted effort is almost as old as the space age itself (Klein *et al.*, 1972) and continues to be a central research question guiding NASA's strategies for space exploration. Much progress has been made to develop the framework for such a search, from identifying the key ingredients for a habitable environment (*e.g*., Capone, 2006; Hoehler, 2007; Shock and Holland, 2007; Lammer *et al*., 2009; Cockell *et al.*, 2016) to expanding and refining biosignature detection strategies (*e.g*., Summons *et al.*, 2008; Nadeau *et al.*, 2016; Benner, 2017; Marshall *et al.*, 2017; Neveu *et al.*, 2018).

Further, strategies are being defined to ensure measurements are robust against false positives and meaningful in the absence of life signatures (Hand *et al.*, 2017; Neveu *et al.*, 2018; National Academies of Sciences, Engineering, and Medicine, 2019). And yet, since *Viking* of the 1970s, no flown NASA missions have been specifically designed with the *primary* goal of searching for evidence of life (present or past). Instead, subsequent planetary missions have focused on searching for and characterizing environments that could support life as a necessary preamble to eventually searching for evidence of life there.

With the discovery of chemolithotrophic ecosystems at the bottom of Earth's oceans and beneath kilometers of ice (Corliss *et al.*, 1979; Horrigan, 1981; Lin *et al.*, 2006), light-independent ecosystems in Earth's crust (Stevens and McKinley, 1995; Chapelle *et al.*, 2002), and sessile communities far from the ice shelf of an Antarctic ice sheet (Griffiths *et al.*, 2021), the subsurface oceans of icy moons around Jupiter and Saturn (*e.g*., Nimmo and Pappalardo, 2016) have become increasingly compelling astrobiological targets (*e.g*., Lunine, 2017; Hendrix *et al.*, 2019). Congress recognized this potential by directing NASA “to create an Ocean World Exploration Program whose primary goal is to discover extant life on another world using a mix of Discovery [∼$500M development-phase cost cap], New Frontiers [∼$1B cost cap], and Flagship [no cost cap, typically >$1.5B]-class missions consistent with the recommendations of current and future Planetary Decadal surveys” (^*^H.R., 2016).

The Roadmap to Ocean Worlds Committee, commissioned by the Outer Planets Assessment Group, proposed a five-part strategy for such discovery: (1) identify Ocean Worlds; (2) characterize their oceans; (3) evaluate their habitability; (4) search for life; and (5) characterize any life we might find (Hendrix *et al.*, 2019).

Enceladus, a 500 km diameter Moon of Saturn, is one such Ocean World. Unlike others, however, materials from Enceladus’ ocean are readily accessible via a persistent plume emanating from its South Polar Terrain (SPT). Ice grains and vapor erupt in jets and/or curtains at velocities typically <0.1–1 km/s from the “tiger stripes” (subparallel, linear fractures approximately 130 km long and 2 km wide) in the SPT (Spencer and Nimmo, 2013 and references therein).

A subsurface liquid body has long been invoked as the ultimate source of the plume material (Porco *et al.*, 2006, 2014; Matson *et al.*, 2007; Schneider *et al.*, 2009; Waite *et al.*, 2009; Postberg *et al.*, 2011) and to explain the observed high heat flux from the SPT (Spencer *et al.*, 2006; Meyer and Wisdom, 2007; Roberts and Nimmo, 2008; Tobie *et al.*, 2008). Gravity field and shape data were consistent with both a regional sea and global ocean (Nimmo *et al.*, 2011; Iess *et al.*, 2014).

The inference of a global subsurface ocean from the fracture history (Patthoff and Kattenhorn, 2011) and a reanalysis of the gravity field (McKinnon, 2015; Hemingway *et al.*, 2019) were confirmed by observations of physical liberation (Thomas *et al.*, 2016) and topographic evidence for true polar wander (Tajeddine *et al.*, 2017).

Simple and complex (*i.e*., greater than ∼100 Da) organic compounds were detected among the water ice and vapor plume material (Waite *et al.*, 2009, 2017; Postberg *et al.*, 2011, 2018; Khawaja *et al.*, 2019). The presence of salts (Postberg *et al.*, 2011), nearly pure silica nanograins (Hsu *et al.*, 2015), carbon dioxide (CO_2_), methane (CH_4_) (Bouquet *et al.*, 2015), and molecular hydrogen (H_2_) (Waite *et al.*, 2017), in the plume suggests that the ocean is geochemically interacting with a rocky core, generating chemical disequilibria. On Earth, seafloor hydrothermal systems that emit CO_2_, CH_4_, and H_2_ can both host *de novo* abiotic organic synthesis (McDermott *et al.*, 2015) and provide chemical energy for some of the most primitive known forms of life (Reveillaud *et al.*, 2016).

We, therefore, have compelling evidence that Enceladus’ ocean meets the canonical requirements for habitability: liquid water, chemical building blocks, and energy sources (*e.g*., McKay *et al.*, 2008, 2014; Cockell *et al.*, 2016; Waite *et al.*, 2017; Postberg *et al.*, 2018; Cable *et al.*, 2021). *Cassini* checked off the first three elements of the Roadmap to Ocean Worlds for Enceladus—the only world to reach this level of characterization. Taking the next steps by searching for signs of life is, therefore, well motivated.

The search for evidence of life at Enceladus is further readily achievable due to the relative accessibility of fresh oceanic material on the surface and from orbit. The plume of Enceladus offers the unique opportunity for studying the subsurface ocean without the need for subsurface access; that is, without the need to descend through at least several kilometers of ice. Both the ejection and fallout of Enceladus’ plume material have been mapped (Spitale and Porco, 2007; Porco *et al.*, 2014, 2017; Helfenstein and Porco, 2015; Spitale *et al.*, 2015; Teolis *et al.*, 2017; Hedman *et al.*, 2018; Southworth *et al.*, 2019), providing new insight into both in-orbit and on-surface sample collection opportunities.

Ground-based Cassini observations over several decades of the plume, Saturn's E-ring, and the paucity of surface craters indicate that the plume is long-lived and modeling suggests that such activity could be sustained over millions to billions of years (*e.g*., Horányi *et al.*, 2008; Kirchoff and Schenk, 2009; Kempf *et al.*, 2010; Choblet *et al.*, 2017; Hansen *et al.*, 2020; Hemingway *et al.*, 2020; Ingersoll *et al.*, 2020).

Although the ejection mechanics governing the plume, and thus the potential for modification of the sample, are uncertain, the detection of organics by Cassini (Postberg *et al.*, 2018a; Khawaja *et al.*, 2019) and recent experimental and modeling efforts quantifying the survivability of organics during high-velocity impacts (Blank *et al.*, 2001; Bowden *et al.*, 2009; Burchell *et al.*, 2014; New *et al.*, 2020, 2021; Jaramillo-Botero *et al.*, 2021) provide confidence that biosignatures could be retrievable from plume samples. Bubble-scrubbing, as well as the process by which bursting bubbles concentrate organic materials at the vacuum-liquid interface, may even concentrate certain signatures (*e.g*., Porco *et al.*, 2017).

As part of a mission concept study commissioned by NASA to evaluate the trades associated with Flagship-class mission architectures for the 2023–2032 Planetary Science and Astrobiology Decadal Survey (conducted by the National Academies of Sciences, Engineering, and Medicine, NASEM), we created a comprehensive science investigation to address the key goals of searching for life at Enceladus and quantifying Enceladus’ habitability. These objectives were used to explore a set of four mission architectures with common science mission objectives and evaluate the science return per dollar.

Critically, we sought to elucidate the science scope of a Flagship-class architecture, given the demonstration of Enceladus exploration with New Frontiers-class mission concepts by previous proposals and studies (*e.g*., MacKenzie *et al.*, 2016; Cable *et al.*, 2017; Eigenbrode *et al.*, 2018). The architecture selected as the best return for investment was one in which a single spacecraft would first orbit Enceladus and then land, called Orbilander. It is detailed in a companion paper (MacKenzie *et al.*, 2021) and a report to NASA, which also describes the other three architectures studied.

Here, we present the rationale by which we derived science objectives from the multitude of questions left to answer at Enceladus (*e.g*., Cable *et al.*, 2021) and arrived at a payload of instrument types. This process is inherently iterative: Requirements for scientific measurements inform which instrument types to select, but instrument and spacecraft capabilities inform which measurements can be accommodated. In this article, we therefore first outline how the science objectives address the primary (Is there life in Enceladus’ ocean?) and secondary (To what extent is Enceladus’ ocean able to sustain life and why?) questions. We then trace these science objectives to a set of measurement requirements to demonstrate how a model instrument payload would meet those requirements, and we conclude with a discussion of the implications for future Enceladus exploration.

## 2. Science Objectives

The search for evidence of life, our primary goal, is directly addressed by the search for potential biosignatures and is supported by the goals of quantifying the habitability of the ocean and understanding the mechanics of ejection that affect the sample between its synthesis and its measurement. The latter provide a crucial context to the search for evidence of life and thus are of high science priority but are considered lower priority in mission architecture decisions. Together, these three goals, summarized in Table 1, represent an appropriately broad Flagship-level scientific scope, providing meaningful understanding into the extent to which, and why, Enceladus is habitable and (perhaps) inhabited.

**Table 1.** Science Traceability Matrix Derived for This Study

Science		Feature of life (from Neveu et al., 2018) or environment	Measurement	Rationale (biosignature/interpretation)	Instrument type
Questions	Objective				
Is Enceladus inhabited?	1. Characterize the bulk organic fraction of volatile and non-volatile plume materials	Potential biomolecule components	1A. Molecular weight distribution of organic matter from 16 Da (CH_4_) to ≥1000 Da in plume vapor and icy particles 1B. Relative abundance and diversity of organic functional groups, including whole molecules, molecular fragments, and compounds potentially indicative of life such as hopanes 1C. ^13^C/^12^C abundances of CO_2_, CO, and CH_3_-type molecular fragments released by heating sampled material at different temperatures	A. Pathway Complexity Index: distribution of the number of types of operations needed to obtain each molecule in the pool of detected organic compounds (Marshall *et al.*, 2017). B. Chemical Context: Inventory of organic chemical species, including specific functional groups, in the context of known biotic and abiotic distributions. C. Isotope Patterns: Isotopes become unevenly distributed due to biological and abiotic processes in ways that inform the history and provenance of the material (Hinrichs *et al.*, 2001).	HRMS; μCE-LIF
	2. Characterize the amino-acid composition of plume materials	Potential biomolecule components	2A. Relative abundances of a.a. isomers, including at least Gly and four of: Ala, Asp, Glu, His, Leu, Ser, Val, Iva, β-Ala, γ-aminobutyric acid, and aminoisobutyric acid, with at least one abiotic and biotic representative, at accuracy ≤10%	Use Gly abundance to normalize other abundances: Gly is the smallest a.a. and the most common in abiogenic meteoritic samples (Creamer *et al.*, 2017). Aminoisobutyric acid is abiotic, rare on Earth, but abundant in some carbonaceous meteorites (Glavin *et al.*, 2020). β-Ala and γ-aminobutyric acid are found in biology as metabolic intermediates but are not used to build proteins (Glavin *et al.*, 2020). The other amino acids are proteinogenic and expected in higher abundance relative to Gly in biogenic samples compared with abiogenic samples. A 10% relative uncertainty enables a clear distinction between biotic and abiotic distributions (Creamer *et al.*, 2017).	SMS; μCE-LIF
		Enantiomeric excess	2B. Relative abundances of l- and d-enantiomers of a.a. with molecular mass b/w d/l-Ala (71 Da) and d/l-Glu (129 Da), including ≥2 among Ala, Val, and β-amino-n-butyric acid; ≥3 proteinogenic; 1 abiotic a.a.; and histidine at accuracy ≤10%	Ala, Val, and β-amino-n-butyric acid are usually present as racemic or near-racemic mixtures in meteorites and other abiotic systems (Glavin *et al.*, 2012, 2020). Histidine has not been found abiotically (Glavin *et al.*, 2020).	
	3. Characterize the lipid composition of plume materials	Potential biomolecule components	3A. Relative abundances, composition, and commonalities of compounds that define subsets of long-chain aliphatic hydrocarbons (*e.g*., carboxylic acids, fatty acids, (un)saturated hydrocarbon chains) up to 500 Da at accuracy ≤20%	Distribution abundance pattern as a function of carbon chain length; patterns in molecular size distributions (*e.g*., limited carbon chain length range) (Summons *et al.*, 2008).	
	4. Search for evidence of a genetic biopolymer in plume materials	Functional molecules and structures	4A. Presence of a polyelectrolyte (polymer with a repeating charge in its backbone)	A linear polymer with repeating charge could be a universal feature of life, indicative of a biological entity capable of Darwinian evolution (Benner, 2017). This can also be used as a terrestrial contamination test, for example, by looking for DNA sequences of microorganisms common in clean rooms.	Nanopore sequencer
	5. Search for evidence of cells in plume materials	Cells	5A. Morphology (size, shape, and aspect ratio) of non-icy particles as small as 0.2 μm in diameter 5B. Organic content (*e.g*., native autofluorescence) associated with non-icy particles	Morphologies resembling cells colocated with physical activity (*e.g*., motion), chemical activity, or biocompositional features.	Microscope
To what extent is Enceladus’ ocean able to sustain life and why?	6–7. Determine the physical–chemical environment of the ocean	Ocean pH	6.1A. Hydrogen ion concentration	Direct measurement.	ESA
			6.1B. Abundances of CO_2_, and bicarbonate or carbonate; relative abundances of all organic and inorganic species (*e.g*., Cl-containing compounds, carbonates, sulfates, metal hydroxides, silica, and silicates)	pH Derived from relative abundances of CO_2_, and bicarbonate or carbonates in the plume (Glein *et al.*, 2015).	HRMS
		Ocean temperature	6.2A. Relative abundances of D/H of H_2_, D/H of H_2_O, and ethylene/ethane 6.2B. Relative abundances of bulk organic and inorganic species (*e.g*., Cl-containing compounds, carbonates, sulfates, metal hydroxides, silica, and silicates) with masses ≤500 Da	A. Derived from geothermometer species (Proskurowski *et al.*, 2006; Fiebig *et al.*, 2013). B. Infer possible reaction temperatures from high-mass species and silica concentration (*e.g*., Sekine *et al.*, 2015).	HRMS
		Ocean salinity	6.3A. Conductivity of plume materials 6.3B. Abundance of Na, Cl ions	A. Direct measurement. B. Direct measurement. Na and Cl appear to dominate Enceladus’ salt abundances (*e.g*., Postberg *et al.*, 2018a).	ESA; HRMS
		Sources of nutrients and energy	6.4A. Presence and relative abundances of CHNOPS-bearing compounds (including H_2_) in plume materials and other micronutrients (*e.g*., Ca, Mg, and Fe) 6.4B. Redox potential (Eh) 6.4C. Abundances of oxidants (*e.g*., SO4–2, CO_2_ or HCO_3_^−^, NO_3_^−^, O_2_) and reductants (*e.g*., H_2_S, CH_4_, NH_3_ or NH4+, H_2_) 6.4D. Presence and relative abundances of products of radiolytic decomposition of surface water ice (*e.g*., OH^−^, H_2_O_2_)	A. Direct measurement. Cassini detected all but P,S. B. Inferred from the presence and abundance of redox-active species. C–D. Constrains redox disequilibria due to products of radiolytic decomposition of surface ice (McCollom and Shock, 1997) by comparing plume abundances with surface deposits (Ray *et al.*, 2021).	HRMS; ESA
		Structure, dynamics, and evolution of the interior	7A. Body-wave arrival times 7B. Tide-induced displacement 7C. Free oscillations	Seismic sources are expected to abound on Enceladus’ fractured SPT, which experiences cryovolcanism and tidal flexing (*e.g*., Kite and Rubin, 2016; Běhounková *et al.*, 2017). Monitoring seismic waves propagated through Enceladus’ interior and along its surface probes its interior structure (ice thickness, ocean depth, core size and mechanical properties, and ocean stratification if any), surface, and shallow fracturing due to tidal flexing, and associated tidal periodicities, informing the properties and processes affecting ocean samples along their path to the plume (Vance *et al.*, 2018).	Seismometer
			7D. Abundances of noble gases (especially ^40^Ar), K, D/H, and ^16^O/^18^O	Determine the abundance of radiogenic ^40^Ar to constrain its timescale of accumulation (Waite *et al.*, 2009). The Cassini INMS measurement could not be reproduced in later observations due to both insufficient sensitivity and mass resolution to distinguish ^40^Ar from organic compound fragments. This also enables radiometric dating of plume material by concurrently measuring the plume K abundance (*e.g*., via MS) (Postberg *et al.*, 2011). Expect a K-Ar age = solar system age, unless Enceladus rock experienced a more recent resetting (*e.g*., melting) event. Determine whether noble gas abundances are similar to or distinct from those expected if trapping into clathrate hydrates occurs, informing the presence of clathrates and their ability to trap other volatiles (*e.g*., H_2_, CO_2_, CH_4_) and affect the thermal and mechanical properties of the interior and the composition of the ocean (Bouquet *et al.*, 2015).	HRMS
	8.1. Characterize the structure and dynamics of the crust	Intracrust fluid reservoirs	8.1A1. Body wave coda, body and surface wave arrival times 8.1A2. Radargrams over SPT	A1. Used to determine the speed of sound and any attenuation within the ice shell due to local fluid reservoirs; detect seismic sources due to fluid flow in the shell (Vance *et al.*, 2018). A2. Dielectric changes indicative of compositional changes result in interfaces that reflect radar-emitted waves (Grima *et al.*, 2016; Heggy *et al.*, 2017).	Seismometer; Radar sounder; Gravity science
		Regional crustal thickness	8.1B1. Surface wave dispersion curves, body and surface wave arrival times 8.1B2. Radargrams over SPT	B1. Seismic monitoring can be used to derive ice shell thickness variations of <50 m with spatial resolution <1 km (Vance *et al.*, 2018). B2. Density changes indicative of compositional changes result in interfaces that reflect radar-emitted waves (Heggy *et al.*, 2017).	
		Regional topography and Love numbers	8.1C1. Limb profiles 8.1C2. Height of surface	Spatiotemporal variations of Enceladus’ shape are essential in helping determine Enceladus’ interior structure (Thomas *et al.*, 2016; Hemingway and Mittal, 2019).	Navigation cameras; NAC; Laser altimeter
			8.1D. Love numbers, hi, *h*_2_, *l*_2_, and *k*_2_ to 0.1%	Temporal variations of Enceladus’ shape and gravity constrain the mechanical properties of the interior (*e.g*., Iess *et al.*, 2014; Hemingway and Mittal, 2019). Meaningful constraints can be derived with sub-percent precision on the Love numbers.	Laser altimeter, Gravity science
	8.2. Infer ascent and freezing conditions	Composition	8.2A. Composition of plume grains at various locations, altitudes, and mean anomalies	Spatiotemporal variations in plume compositions constrain processes and conditions of eruption. The kinetics of freezing during ascent can influence composition (Thomas *et al.*, 2019). The plume composition varies with tidal forcing (Hurford *et al.*, 2007) and spatially (grain-to-gas ratio; Hedman *et al.*, 2018), finer compositional variations remain to be elucidated.	HRMS; ESA
		Rate of fallout	8.2B. Rate of plume material collected in orbit and on the surface	Quantify the mass of ejected material that cannot be sampled during fly-throughs.	Fallout collector; particle counter
	8.3. Determine the physical structure of the jet vent openings	Surface thermal properties	8.3A. Thermal emission spectra at wavelengths 10–50 μm	Map surface temperatures, surface heat flux, and determine the thermal properties of surface material from temperature variations (*e.g*., Howett *et al.*, 2011).	TES
		Vent morphology and topography	8.3B. Surface topography near the vents at sub-meter horizontal, 10 cm vertical resolution	The topography near vents informs their shape and thus conditions (*e.g*., temperature, pressure, velocity) encountered by ocean samples during ejection (Goldstein *et al.*, 2018; Ono *et al.*, 2019).	NAC; laser altimeter
			8.3D. Horizontal and vertical surface displacement at sub-meter spatial resolution, 10 cm vertical resolution	Tidally modulated vent eruptions (Hurford *et al.*, 2007; Goldstein *et al.*, 2018) expected to result in m-scale displacement (Běhounková *et al.*, 2017) quantifiable with dm-scale resolution.	Laser altimeter
		Subsurface structure	8.3C. Location and extent of liquid-filled pockets in the SPT	See 8.1A.	Radar sounder; seismometer

The science objectives listed here are those that drive requirements and are therefore not an exhaustive list of all science objectives that could be done at Enceladus or even with this example payload.

μCE-LIF = microcapillary electrophoresis with laser-induced fluorescence; a.a. = amino acid; CH_4_ = methane; CO_2_ = carbon dioxide; ESA = electrochemical sensor array; H_2_ = molecular hydrogen; HRMS = high-resolution mass spectrometer; NAC = narrow-angle camera; SMS = separation mass spectrometer; SPT = South Polar Terrain; TES = thermal emission (imaging) spectrometer.

### 2.1. Search for evidence of life beyond Earth

There are numerous and diverse approaches to searching for evidence of life that can be implemented on spaceflight missions (*e.g*., Neveu *et al.*, 2018). Given constrained mission resources of mass, power, and data return, we prioritized the search for chemical signatures of life (organic compounds of biological origin) for several reasons. First, *Cassini* confirmed organic compounds in the plume material, so measurements that require further analyses of organics are lower risk (*e.g*., *Cassini* data can directly inform instrument selection and design). Second, as captured in the NAS document “An Astrobiology Strategy for the Exploration of Mars” (2007) “… of all the various life-detection techniques available, analysis of carbon chemistry is the first among equals.”

This is also true for Enceladus, both in terms of the diversity of complementary organic chemical analyses that can be implemented, and in terms of their sensitivity. Third, organic chemical analyses, when properly designed and executed, can provide a broad context to a potentially negative result, for example, by providing insight into abiotic or prebiotic chemistry. Finally, several technologies relevant to organic chemical analyses are currently available for infusion into flight (Technology Readiness Level, TRL, 6 or higher) that can make complementary and/or repeated measurements, making the results more robust against instrumental false positives (by adding complementarity) or malfunction (by adding redundancy).

Recognizing that finding unambiguous evidence of life will require multiple, independent lines of evidence (*e.g*., Neveu *et al.*, 2018), the proposed life detection strategy includes seven distinct investigations, including two higher-risk but higher-reward measurements: a search for a linear polyelectrolyte; and a search for cell-like structures. It should be emphasized that none of the “life-detection” measurements discussed here can be considered in isolation. The final interpretation of whether Enceladus is inhabited or not would be based on the collective analysis of all the data, including the contextual information.

One of the main motivations to search for evidence of life at Enceladus is access to fresh ocean material in the plume (and in the plume fallout). However, in orbit and on the surface, plume materials would be sampled only seconds after they are ejected from the interior. Since ultraviolet (UV) and ionizing radiation at Saturn's moons, including Enceladus, are relatively benign compared with other solar system targets such as the jovian moons (*e.g*., Nordheim *et al.*, 2017, 2018), alteration of organic matter and potential molecular biosignatures during plume ejection and before sampling is expected to be limited.

In the following sections, we highlight different life-detection measurements that could establish the origin (biotic or abiotic) of organic matter in the plume (Sections 2.1.1–2.1.3), and measurements that could provide insights into the nature of forms of life in the ocean if present (Sections 2.1.4–2.1.5).

#### 2.1.1. Molecular Assembly Index

Biochemistry on Earth facilitates and relies on the creation of a variety of complex organic molecules. Thus, organic molecule complexity has been put forth as a potential biosignature (Marshall *et al.*, 2017), though how complexity is defined and quantified remains a topic of active research. One compelling framework for quantifying organic complexity and establishing an abiotic/biotic threshold is the Molecular Assembly (MA) Index put forth by Marshall *et al.* (2021). These authors used a database of organic molecules of up to 800 Da to demonstrate that the assembly theory (the distribution of operations and the number of types of operations needed to create each molecule) could be used to distinguish abiotic from biotic sources, without assumptions about the nature of the processes at work.

Thus, this metric is agnostic as to the kind of organic molecules produced by life that may exist on a planetary body and is particularly powerful when coupled with contextual knowledge about the geochemical pathways available.

A survey of the plume particulate content with a mass spectrometer that is capable of isolating ions of individual molecules from complex mixtures and with adequate sensitivity (∼ppb) over a mass range of 250 Da to at least 800 Da could provide the data necessary to determine the MA Index as was verified in Marshall *et al.* (2021). The MA is a computed number or index based on the smallest number of assembly operations it would take in mathematical space to construct a final molecule from its basic molecular sub-units, where each sub-unit is structurally different. Using laboratory and environmental samples and tandem mass spectrometry (MS/MS) to isolate individual molecular spectra above 250 Da, Marshall *et al.* (2021) determined that MA values of 15 or more reliably infer a biological source.

Thus, since fragmentation products of a parent ion can be correlated with the MA, identifying compounds and their daughter products would be necessary. The distribution of estimated MA values from these measurements can then be compared with abiotic, dead, inorganic, and biotic distributions: Only biological processes produce high MA values. The broad nature of mass spectrometer measurements also offers the opportunity for fortuitous science return by facilitating the identification of other potential biomolecules or environmental indicators such as saccharides that are not specifically targeted.

#### 2.1.2. Amino acid characterization

The detection of amino acids alone is not necessarily a biosignature, given that they can be created from both biotic and abiotic processes (*e.g*., Koga and Naraoka, 2017). However, there are at least three attributes of biologically derived amino acids here on Earth that can be exploited to discern abiotic or biotic origin (Glavin *et al.*, 2020): abundance pattern, chiral excess, and isotopic fractionation.

First, a pattern in the relative abundance of amino acids can be a distinguishing metric (McKay, 2004; Dorn *et al.*, 2011). The relative concentrations of amino acids derived from biotic sources reflect life's preference for specific molecules based on the functional roles they can bestow in proteins. In contrast, the relative concentrations of amino acids derived from abiotic sources are dictated by reaction kinetics and thermodynamics; they tend to exhibit specific patterns dominated by small, low-formation-energy molecules formed from simple chemical processes (Cobb and Pudritz, 2014).

Second, a large excess of one enantiomer (l or d) in a diverse set of amino acids can also be an indication of preferential chiral synthesis or degradation, implying biological sources using chiral molecules. Terrestrial life exclusively uses l-**α**-amino acids to form peptides and proteins whereas abiotic processes tend to form racemic mixtures where the l- and d- forms are almost equally represented. However, homochiral mixtures of **α**-amino acids tend to become racemic spontaneously over time and an excess of more than 20% up to 60% of the l-form has been measured in a few meteoritic **α**-amino acids (*e.g*., Glavin *et al.*, 2012). In fact, some bacteria also use d-**α**-amino acids in their cell walls (*e.g*., Zhang and Sun, 2014), including deep-sea microorganisms (Kubota *et al.*, 2016; Wang *et al.*, 2020). Therefore, caution must be taken when interpreting enantiomer asymmetries in amino acids. On the other hand, a large excess of d-amino acids (a condition not yet measured in abiotic systems) might be a compelling indicator of a biological origin of life distinct from Earth's.

Finally, biogenic amino acids tend to be enriched in the lighter isotopes, creating an imbalance in the ratios of ^13^C/^12^C, ^15^N/^14^N, and D/H. Together, these three measurements provide a more robust approach to assert the origin of amino acid mixtures than any one of them, as recently discussed in detail by Glavin *et al.* (2020). However, given the relative difficulty of measuring compound-specific isotopic abundances with current high-TRL instrumentation (though technologies are advancing, see Arevalo *et al.*, 2018), we considered only measurements of the relative abundance of amino acids and enantiomeric excess as science objectives in this mission concept study.

#### 2.1.3. Lipid hydrocarbon characterization

Lipid compounds are also created from both abiotic and biotic processes. Earth-life uses phospholipid bilayers for self-enclosure (membranes) and to regulate chemical exchanges with the environment via peripheral and integral proteins embedded within the bilayer. Functional lipid membranes in Earth life are made of amphiphilic lipids, with an aliphatic hydrocarbon chain providing hydrophobicity, and a highly polar phosphate group. It is assumed that lipid membranes will be common in water-based life (Georgiou and Deamer, 2014).

Environmental characteristics have driven the optimization of membrane fluidity/rigidity of microorganisms’ phospholipid membranes via factors such as the presence of the double bonds (*i.e*., whether the hydrocarbon chains are saturated or unsaturated) and the length of the hydrocarbon chains. Hydrocarbon chains that are too long cause membranes to be too rigid. Hydrocarbon chains that are too short cause membranes to be too fluid or unstable.

This need for tight regulation of the hydrocarbon chain length is reflected in at least two attributes of membrane-forming lipids that can be exploited to discern a biotic source (Summons *et al.*, 2008; Georgiou and Deamer, 2014). (1) Cell membranes are typically built with C12 to C30 lipid hydrocarbons (*e.g*., Eigenbrode, 2008). At physiologically relevant temperatures, hydrocarbon chains shorter than C12 can become volatile, whereas hydrocarbon chains longer than C30 can become too waxy. In the cellular membranes of Earth-life, C16–C18 lipid hydrocarbons appear at the highest abundance, and the relative abundance of hydrocarbon chains diminishes toward longer and shorter lengths.

In contrast, hydrocarbon chain lengths in abiotic systems are strongly skewed toward short chain lengths (<C8), with an exponential decay in molecular abundance as a function of chain length (Lawless and Yuen, 1979; Shimoyama *et al.*, 1986; Naraoka *et al.*, 1999). Long chains (>C12) are rarely produced by abiotic processes, or they are produced at relatively low abundances. (2) Lipid biosynthesis in Earth-life proceeds through the addition of specific subunits (C2-acetate and C5-isoprene) to the hydrocarbon chain. This leads to a higher predominance of even or odd chain lengths (due to C2-additions and C5-additions, respectively) that is not observed in abiotic systems (Summons, 2008).

Lipid hydrocarbons are an attractive target for life detection because they can persist in the environment over geologic time, being resilient to degradation by, for example, heat or water (Eigenbrode, 2008). To detect lipid hydrocarbons and discern structural and abundance patterns, the relative molar abundance of molecules up to at least 500 Da, or their fragmentation products, must be determined with ≤20% accuracy. Isotopic measurements can add strength to the interpretation of a lipid distribution pattern—biologically derived lipids would be expected to have lighter isotopes (Horita and Berndt, 1999)—but are not included in this study's measurements due to a lack of high-TRL instrumentation at the time this concept study was performed.

#### 2.1.4. Polyelectrolyte search

A polyelectrolyte chain, a linear polymer with a repeating charge, is postulated to be a universal feature of life indicative of biological entities that are capable of Darwinian evolution (Benner, 2017). Life on Earth universally uses DNA/RNA, but other chemical and structural solutions are possible (Eschenmoser, 2004). Depending on the detection method, this potential biosignature can be agnostic to the biochemistry at work (Pinheiro *et al.*, 2012). The search for a polyelectrolyte chain is high risk/high reward, and therefore an excellent complement to other chemical experiments that focus on amino acids and lipids, which are more likely to be present even in the absence of life.

The high risk stems from the relative instability of these types of molecules, their expected low abundances (see Section 4), our *a priori* lack of knowledge of their structure and composition, and the potential for terrestrial contamination (The latter can be mitigated by using in-flight decontamination steps, and it can also be screened based on genetic matches to DNA/RNA sequences in microorganisms commonly found in clean room facilities and on spaceflight hardware; Venkateswaran *et al.*, 2001).

The potential for a high reward stems from the unambiguous biological interpretation of a positive detection (assuming possible contamination sources are ruled out) and from the types of information that could be extracted about these molecules on further characterization of the returned data. The latter includes the biochemical underpinnings of the organisms that synthesized them (*e.g*., the genetic alphabet) and new critical insights of the processes that lead from abiotic chemistry to the first replicating organisms.

Extracting that information may require a follow-up sample return mission to exploit more sophisticated laboratory technologies, but the discriminatory power of *in situ* detection of a polyelectrolyte chain, in addition to its potentially high scientific return, drove us to prioritize the search for polyelectrolytes over the characterizations of possible cells (described in Section 2.1.5).

The technology exists for detecting the presence of polyelectrolyte chains, and even potentially sequencing the encoded genetic information, but is not yet flight-qualified (Carr *et al.*, 2017). The MinION^™^ nanopore sequencer, for example, has been successfully operated on the International Space Station (Castro-Wallace *et al.*, 2017) and parabolic flights of varying simulated gravity (Carr *et al.*, 2020), but it relies on biological pores that would degrade over long-lived missions.

The Enceladus Orbilander mission concept, for example, would require 7 years of cruise to reach the Saturn system and a further 4.5 years to pump down enough energy to enter Enceladus orbit (MacKenzie *et al.*, 2021)—a duration much longer than the 12-week useful lifetime quoted for the MinION by the manufacturer. Further, biological pores are tuned for the biochemistry of Earth: Proteins bind specifically to nucleic acids to ratchet the molecules through the pore. Life is expected to arise independently (if at all) in the outer solar system and thus may not use the adenine, guanine, thymine, uracil, and cytosine of terrestrial DNA to build its informational carriers.

Synthetic nanopore systems are in development through NASA-funded programs such as PICASSO and COLDTech and also through commercial and academic enterprises (*e.g*., Xue *et al.*, 2020 and references therein). Notably, a solid-state nanopore sequencer is the least flight-ready (*i.e*., lowest TRL) instrument required to meet the science objectives defined here. Although the multiple, ongoing development efforts will hopefully result in nanopores of sufficient TRL for a mission launch before the end of the 2030s (the concept study's baselined launch date), there is always an inherent risk when an instrument requires significant development.

We, therefore, note that the science objectives described here are specifically designed to be robust to such an eventuality. That is, a delay in nanopore sequencer development would not represent a death knell to the search for evidence of life at Enceladus: A search for an evidence-of-life mission at Enceladus is compelling without a nanopore sequencer. The search for cells with a microscope, described in the following section, also represents a non-chemical potential biosignature and can, therefore, serve as the “confirmation” biosignature.

#### 2.1.5. Cell search

Identifying an intact cell can represent a strong indicator of life, orthogonal to the chemical indicators listed earlier. That identification, however, cannot rely on morphology alone. Several additional cellular attributes are measurable with spaceflight instrumentation, such as motion, autofluorescence, fluorescence, and biomechanical properties (Nadeau *et al.*, 2008, 2018).

However, like the search for a polyelectrolyte, the high reward potential of a search for cells comes with an element of risk. For this objective, the risk lies in the uncertainties in ejection mechanics and biomass available in the plume. Measuring motion and/or biomechanical properties require that sampled cells remain alive after their journey from the ocean to space (if sampled in the plume, *e.g*., Porco *et al.*, 2017) and on the surface (if sampled there). The likelihood of capturing viable cells is only beginning to be constrained.

Bywaters *et al.* (2020), for example, show that a few percent of *Escherichia coli* survive transport through a pressurized nozzle into vacuum, and Cosciotti *et al.* (2019) documented the survivability of cyanobacteria in low temperatures and salinities expected for Ocean Worlds. For the purposes of this study, we chose to avoid the requirement of a viable, motile cell by targeting only morphology and autofluorescence (Bhartia *et al.*, 2010; Hand *et al.*, 2017), though future work should investigate which dyes might be included to conduct a more encompassing fluorescence investigation. Tailoring to the water-rich, mineral-poor environments of ocean world exploration will certainly yield different formulations than fluorescence campaigns targeted for the search for evidence of life on Mars (*e.g*., Yamagishi *et al.*, 2018).

#### 2.1.6. False negatives/positives and null results

The strategy outlined earlier to search for evidence of life at Enceladus seeks to minimize the likelihood of a false negative result. As recommended in the document “An Astrobiology strategy for the Search for evidence of Life in the Universe” (NASEM, 2019), multiple, independent lines of evidence coupled with a detailed environmental context can help mitigate the likelihood of false negative results. This includes the motivation to search for seven independent but complementary potential signs of life at Enceladus, and to also obtain detailed physicochemical information of the ocean (Section 2.2.1), as well as a better understanding of the internal structure of the Moon (Section 2.2.2) and how ocean materials are ejected into space (Section 2.2.3).

The diversity of independent but complementary measurements also seeks to provide a context for a null (no signs of life found) or an ambiguous result. Some of the signs of life targeted by the Orbilander mission concept are less definitive in isolation. Others, polyelectrolyte chains for example, would, if present, constitute unambiguous evidence of life (provided that a false positive from terrestrial contamination can be ruled out, as explained later). The definiteness of polyelectrolyte chains resides in the improbability of the non-biological processes producing them.

The downside of searching for such unambiguous signs of life is that a null result provides limited information regarding the extent of abiotic/prebiotic organic chemical evolution in the ocean, which is also of great scientific interest. That is why in addition to definitive signs of life, the Orbilander mission concept would target compounds that are expected to be present even if life never evolved on Enceladus, such as lipids and amino acids. In the absence of life signatures, the presence, abundance distributions, and molecular qualities of those compounds would provide insights regarding the extent of organic chemical evolution at Enceladus.

Those insights would inform models of prebiotic chemistry applicable to Earth, and they would contribute to a better understanding of the conditions that might have led to the origin of life on our planet. Targeting multiple compounds (and multiple molecular qualities of those compounds) would also help interpret ambiguous results possibly arising from mixtures of abiotic and biotic sources, or from degradation pathways of organic matter before sampling, noting, for example, that lipid compounds tend to be more resistant to degradation than amino acids or polyelectrolyte chains. Finally, targeting multiple compounds also retires some of the risk associated with uncertainties regarding the potential biomass in the ocean (that uncertainty is also mitigated by accessing different sample pools, and obtaining larger sample volumes on the surface, see Section 4.1).

Planetary Protection guidelines define the levels of spacecraft bioburden required to prevent the forward transfer of viable microorganisms to Enceladus. Orbilander would be a Category IV mission (as per the Committee on Space Research planetary protection designations^*^) and spacecraft bioburden levels would have to be at least as low as those designated for Category IV Mars missions (3 × 10^5^ spores in total; an average of <300 spores per square meter of exposed external and internal spacecraft surfaces) or lower. For a detailed description of forward contamination considerations, see Neveu *et al.* (*Astrobiology*, in review).

However, these limits are superseded in a search for an evidence-of-life mission such as Orbilander, where potential false positives due to terrestrial contamination must also be considered. Contamination must be controlled down to, in some cases, molecular levels to avoid interfering with analyses of samples. Given the low abundance of compounds of interest expected at Enceladus, and given the sensitivity of the analytical payload, the requirements to control molecular contamination are likely more stringent than the requirements imposed on bioburden levels, and they will also require cleaning and validation strategies that are different from those employed to limit bioburden (Eigenbrode *et al.*, 2021).

Samples obtained from sensitive spacecraft surfaces (*e.g*., along the sample chain) to validate contamination levels during assembly, test, and launch operations will also provide an important control for the interpretation of data obtained during the science phase, particularly in the event of a positive result from the life detection experiments. Contamination control would have to be an integral part of the design of a mission such as Orbilander through all mission phases. Possible contamination control strategies include, in addition to the assembly of parts and payload elements in ultra-clean rooms, the use of a full spacecraft barrier, and on-cruise bake-out of critical surfaces (*e.g*., the sample acquisition systems), both of which have proven efficient at reducing particle and molecular contamination by a factor of 10^−3^ and 10^−1^, respectively (Eigenbrode *et al.*, 2021).

### 2.2. Quantify the habitability of Enceladus’ ocean

*Cassini* already established that Enceladus’ Ocean meets the minimal requirements for habitability (NAEM, 2018), thereby providing the evidentiary basis for a follow-up life detection mission (*e.g*., Hendrix *et al.*, 2019). Significant improvements over *Cassini* measurements and new insights into Enceladus’ interior structure would enable major advances in quantifying the habitability of a subsurface ocean environment, beyond a binary yes/no assessment. Included among these improvements is the goal of narrowing by orders of magnitude the current uncertainty on how much biomass Enceladus can support (Cable *et al.*, 2020).

This greater fidelity would be achieved by constraining the types and abundances of Gibbs free energy available in the environment that biology could use for metabolism (*e.g*., Ray *et al.*, 2021), by constraining the extent and style of water–rock reactions through geochemical analysis of ocean materials, and by constraining the distribution of interfaces at which biomass may be more highly concentrated (*e.g*., through seismic analysis). Providing the “why,” “why not,” and “how likely” to a life detection result from an environmental perspective, the following objectives target key contextual evidence supporting the primary life-detection goal.

#### 2.2.1. Physical/chemical environment of the ocean: hydrothermal conditions

**FIG. 1.**
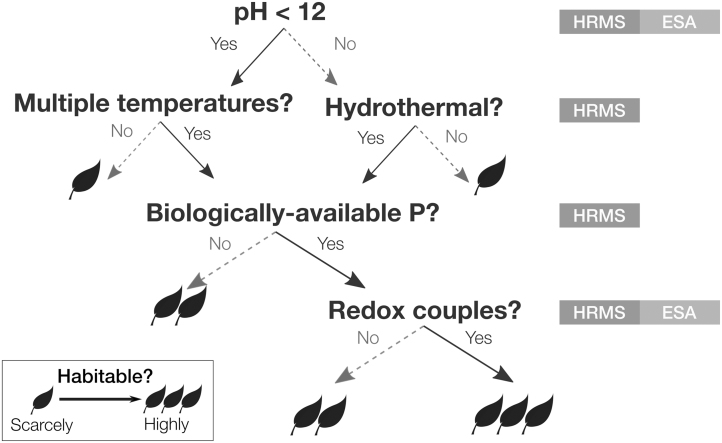
Decision tree demonstrating the benefit of geochemical context for interpreting biosignature results. “HRMS” and “ESA” refer to two notional payload elements (high-resolution mass spectrometer and electrochemical sensor array, respectively) baselined in the concept study to measure the depicted quantities.

Quantifying the habitability of the ocean (a separate question from whether it is inhabited) requires measurements of the physical and chemical state of the environment. Together, these measurements represent a rich scientific investigation that promises to provide the first comprehensive physiochemical picture of an extraterrestrial ocean, with information on par in many cases with what is known about the Earth's deep ocean and water bodies lying beneath the Earth's polar ice sheets. A partial list of the parameters to be determined is shown in Fig. 1, organized as an example flowchart for assessing the habitability of the ocean.

The determination of parameters such as hydrothermal temperatures and pH is accomplished by measuring sets of neutral and ionic species containing salts (Na, K, Mg, Ca) and the elements CHNO, all of which serve as geothermometers and inputs to thermodynamic models of carbon speciation (*e.g*., Giggenbach, 1988; Glein, 2015; Glein and Waite, 2020). The extent of hydrothermal chemistry is provided by such additional species and the measurement of ^40^Ar (McKinnon, 2010). Insight into the evolution of the hydrothermal system comes from comparing ^40^Ar, D/H, K, and ^16^O/^18^O to baseline values and trends set by measurements of possible sources (*e.g*., cometary ices) and processes (*e.g*., volatile encapsulation in clathrate hydrates, or gas hydrate self-preservation, Boström *et al.*, 2021).

Assessing the bioavailability of key elements requires completing the CHNOPS inventory, especially the molecular carriers of P and S. Abundances of many pairs of reduced and oxidized species commonly found in metabolic processes on the Earth are measured to determine the redox state of the ocean relative to chemical equilibrium (*e.g*., Shock and Holland, 2007). This enables the calculation of chemical affinities for specific metabolic reactions, which can be used to both quantify the biomass that can be supported by a given metabolism (Cable *et al.*, 2020) and assess the contribution of biological activity to the chemical state of the ocean (Waite *et al.*, 2017). (The extent to which any of these geochemical measurements is affected by the pathway from ocean to space is addressed by the objectives in Section 2.2.3.)

#### 2.2.2. Physical/chemical environment of the ocean: structure and dynamics of the interior

Understanding the structure and dynamics of the rocky interior via seismic and gravity-field investigations (*e.g*., Vance *et al.*, 2018) provides key insights into the spatial extent and longevity of geochemical interactions. For example, a porous core provides a larger interface area for water–rock interactions and has implications for internal dissipation and the conditions necessary to sustain a global ocean (*e.g*., Neumann and Kruse, 2019).

Determining the structure and dynamics of the ice shell helps quantify the mechanisms and timescales of transport of oxidized species from the surface into the ocean that may act together with burial under plume fallout (million-year timescales = kilometer-scale shell thickness/millimeter-per-year fallout rates) (Southworth *et al.*, 2019). Gravity field, radar sounding, and seismic investigations can provide observational constraints (*e.g*., Vance *et al.*, 2018, Marusiak *et al.*, 2021). Specifically, measuring the tidal Love number *k*_2_ to better than 0.1% would help in detecting tidal lag and constraining the extent of tidal dissipation in the interior (Ermakov *et al.*, 2021).

Measurements of the relative numbers *h*_2_ and *l*_2_ to the same accuracy would help disambiguate the interpretation of the internal rheology. If the ice–ocean interface is detected by radar, characterization of its basal properties may further indicate regions of melting or freezing (Grima *et al.*, 2019). A seismic investigation should be capable of detecting waves generated by strike-slip motions in the ice and possibly also noise generated at the ice–ocean interface by turbulent fluid flows (*e.g*., Panning *et al.*, 2017; Marusiak *et al.*, 2021).

Detecting waves generated in the rocky interior may prove difficult, given the unique problem in Ocean Worlds that the global ocean filters out the shear components of seismic waves, but both gravity and seismic measurements would be useful in detecting an inferred porous region in the upper part of the rocky interior. Seismology might also be used to investigate stratification in the ocean, which would further constrain ocean salinity and whether the values inferred from plume measurements reflect the bulk ocean composition (Lobo *et al.*, 2021; Zeng and Jansen, 2021). Seismic propagation in Enceladus, including waves from the deeper interior, is an active area of study (Vance *et al.*, 2020; Marusiak *et al.*, 2021), but Enceladus’ seismic activity should be more observable than events on Earth's Moon due to the icy satellite's small size and short tidal cycle (orbital period of 33 h).

#### 2.2.3. Investigate plume ejection mechanisms

The presence of water–rock interaction products in the ice grains (Postberg *et al.*, 2011; Hsu *et al.*, 2015; Waite *et al.*, 2017) proves that the ultimate source of the plume material at Enceladus is the subsurface ocean. Diverse compositions of plume grains coming from this single reservoir suggest that compositional enrichments or depletions are at play, for example, due to phased changes such as vaporization at the top of the water table with possible aerosolization, sublimation from grains, and condensation of vapors (Bouquet *et al.*, 2017; Khawaja *et al.*, 2019). Constraints on the path inform how we infer ocean conditions and the nature and composition of ocean material from the measurements of plume material.

The path between ocean and surface, however, remains ill-constrained, arising from uncertainties in which conditions within the ice shell (such as thickness, sources of stress, the extent to which fissures are filled) and the ocean (such as state of overpressure) control fracture initiation, propagation, and longevity (*e.g*., Schmidt *et al.*, 2008; Kite and Rubin, 2016; Spencer *et al.*, 2018; Hemingway *et al.*, 2020). These science objectives address how representative the plume material is of the ocean, and how to account for any ejection-driven changes to ocean material.

Note that alterations of organic matter and potential molecular biosignatures are not in the plume or on the surface. The radiation environment—both ionizing and UV—at Enceladus is relatively benign. For example, in the conservative case that Enceladus’ magnetospheric radiation environment is the same as Mimas (Choukroun *et al.*, 2021), the time to reach a chemically significant dose at 1 mm depth is 100,000 years (Nordheim *et al.*, 2017), which is 2–3 orders of magnitude longer than at Europa (Nordheim *et al.*, 2018).

Extrapolating the wavelength-dependent photolysis rates of glycine to the photon spectrum at Enceladus, Choukroun *et al.* (2021) showed that degradation remains <10% within 1 mm of ice in 3 years. Comparing these timescales with the time from ejection to sample collection suggests that processes within the plume are minimal compared with potential vent processing. For orbital altitudes of 20–60 km, the largest particles travel at ∼200 m/s (Degruyter and Manga, 2011) and thus travel a few minutes before collection.

Catching the larger particles that fall out onto the surface (>10 μm) occurs in even less time, on the order of tens of seconds based on the model of Degruyter and Manga (2011). Particles that reach the surface are quickly buried by other particles (0.01–1 mm/year, depending on particle size) (Southworth *et al.*, 2019), providing shielding from incident radiation, and are only mechanically modified (*e.g*., by sintering) in the warmest surface environments very near the vents (Choukroun *et al.*, 2020).

Establishing whether fluid reservoirs or structural heterogeneities exist within the ice shell would indicate whether the plume plumbing includes pockets or sills where oceanic material can concentrate (*e.g*., due to partial freezing). From buoyancy arguments, fractures are expected to be filled up to a few hundred meters below the surface (Kite and Rubin, 2016). Radar sounding can reveal these structures similar to interrogations of terrestrial glaciers and ice shelves (*e.g*., Blankenship *et al.*, 2009; Schroeder *et al.*, 2020) and will be used to interrogate the jovian moons on upcoming missions *Europa Clipper* and *JUICE* (*e.g*., Bruzzone *et al.*, 2013; Heggy *et al.*, 2017). The thickness of the crust, especially at the SPT, defines the minimal path length for a conduit between the ocean and surface, and it can be determined from radar sounding and seismic monitoring (*e.g*., Kalousová *et al.*, 2017; Vance *et al.*, 2018).

By analogy with geysers and other subsurface fluid motions on Earth, seismic data carry information about the amounts of materials, their speed, and fluid-to-gas transitions occurring during ascent from the ocean. Active and passive electromagnetic measurements would offer similar advantages (Marusiak *et al.*, 2021). Measuring the surface expression of the vent structures—morphology, topography, and thermal properties—conveys information about the venting mechanics (*e.g*., Nimmo and Pappalardo, 2016) and associated conditions encountered by the sample (*e.g*., temperatures, pressures, velocity vectors, compositional interfaces). For example, measurements of thermal emission and vent outlet width (*e.g*., Goguen *et al.*, 2013) and flux of water vapor (Hansen *et al.*, 2011, 2017) provided constraints for fluid dynamic models to derive crack width and extent (Ingersoll and Pankine, 2010; Nakajima and Ingersoll, 2016).

In addition to facilitating the search for biosignatures, measurements of the composition of plume ice grains and vapor as a function of altitude and Enceladus’ mean anomaly enable investigations of whether kinetics of freezing influence composition (Thomas *et al.*, 2019), as well as how orbit controls the plume activity (Ingersoll and Ewald, 2017; Ingersoll *et al.*, 2020).

## 3. Traceability to the Science Payload

We trace these science objectives to a model payload grouped into three suites: the Life Detection Suite (LDS) (Table 2), the Remote Sensing and Reconnaissance Suite (Table 3), and the *In Situ* Suite (Table 4). We selected a suite of instruments with some overlapping capabilities to ensure robustness of the science investigation at the level expected for a Flagship-class mission. The model payload shown here proves that a robust search for evidence for life at Enceladus is possible in the next decade with a reasonable Flagship-class budget. Other implementations are possible and could be explored.

**Table 2.** Instrument Characteristics Used to Model the Life Detection Suite

Item	HRMS	SMS	ESA	μCE-LIF	Microscope	Nanopore	Units
Size/dimensions	0.39 × 0.39 × 0.39	0.15 × 0.25 × 0.12	0.1 × 0.1 × 0.2	0.15 × 0.15 × 0.15	0.11 × 0.2 × 0.1	0.14 × 0.22 × 0.15	m × m × m
Mass with 30% contingency	26	15.6	3.9	4.68	3.9	5.2	kg
Average power with 40% contingency	98	91	21	8.4	21	7	W
Data volume over prime mission with 30% contingency	8.424	1.9188	0.3276	0.00234	0.3861	15.6	Gb

**Table 3.** Instrument Characteristics Used to Model the Remote Sensing and Reconnaissance Suite

	NAC	Wide-angle camera	Thermal emission spectrometer	Laser altimeter	Radar sounder	Units
Size/dimensions	0.39 × 0.39 × 0.70	0.78 × 0.56 × 0.44	0.18 × 0.18 × 0.13	0.26 × 0.28 × 0.28	1.4 × 2.0 × 0.025	m × m × m
Instrument mass with 30% contingency	26	0.52	4.94	9.62	15.6	kg
Instrument average payload power with 40% contingency	7	3.50	18.20	23.1	35.00	W
Instrument average science data rate with 30% contingency	5447	5447	1505	13	10,400	kbps
Instrument fields of view	0.293	44.003	1	0.02	n/a	Degrees

**Table 4.** Instrument Characteristics Used to Model the *In Situ* Suite

		Units
Context Imager		
Size/dimensions	0.38 × 0.25 × 0.15	m × m × m
Instrument mass with 30% contingency	5.2	kg
Instrument average payload power with 40% contingency	16.52	W
Instrument mission data volume with 30% contingency	7.02	Gb
Instrument field of view	15 per lens	Degree
Seismometer		
Size/dimensions	0.075 × 0.075 × 0.045	m × m × m
Instrument mass with contingency (CBE+Reserve)	6.50	kg
Instrument average payload power with 40% contingency	5.6	W
Instrument average science data rate with 30% contingency	0.46	kbps

### 3.1. Life Detection Suite

A high-resolution mass spectrometer (HRMS) would conduct the volatile organic and inorganic characterization for Sci. Obj. 1, 6, 7, and 8.2 (Table 1). The modeled instrument type is a time-of-flight (TOF) mass spectrometer that separates ions by their transit time through a multi-bounce ion optical system. Advantages include high mass resolution (m/Δm >20,000) and a wide mass range up to 1000 Da. A gas inlet with a cryotrap would facilitate sampling the vapor during plume flythroughs. Interior to the spacecraft, the HRMS would also receive vapor from the ice particle Sampling System (SS).

Some recently flown or in-development HRMS include MASPEX on *Europa Clipper* (Brockwell *et al.*, 2016) and COSAC on *Rosetta Philae* (Goesmann *et al.*, 2007). Other HRMS instruments in development for flight include the Orbitrap (Denisov *et al.*, 2012; Briois *et al.*, 2016; Arevalo *et al.*, 2018; Selliez *et al.*, 2019, 2020), multi-turn TOF mass spectrometers (Toyoda *et al.*, 2003), and miniature quadrupole ion mass spectrometers with chemical ionization (Waller *et al.*, 2020).

A separation mass spectrometer (SMS) would characterize simple and complex molecules, including amino acids (relative abundance and enantiomeric excess) and lipids for Sci. Obj. 2 and 3. We selected a gas chromatograph mass spectrometer (GCMS) as the model instrument type due to its high TRL: *Curiosity* SAM (Mahaffy *et al.*, 2012), *ExoMars* MOMA (Goesmann *et al.*, 2017), and *Dragonfly* DraMS (Trainer *et al.*, 2018) all include GC capabilities.

After heating a sample or applying a derivatization agent to it, volatilized materials would be passed through a capillary column to separate compounds by their retention time (related to molecular mass and polarity) at high enough precision to distinguish a full range of organic compounds, including the separation of enantiomeric mixtures. Other approaches, such as augmenting a GCMS with capillary electrophoresis, coupled to the same MS, are in development (PI Brinckerhoff, 18-ICEE2_2-0044) (Creamer *et al.*, 2017).

Separation of individual compounds by liquid chromatography before their identification by MS (liquid chromatography–mass spectrometry [LC-MS]) is also under investigation (Getty *et al.*, 2013; Southard *et al.*, 2014), although the difficulties associated with the handling of such liquids in space environments make this technique of lower TRL. Critically, MS/MS with LC-MS has been demonstrated as specifically enabling for determining MA Index (Marshall *et al.*, 2021), though other separation techniques may also be applicable.

An electrochemical sensor array (ESA) would characterize the physicochemical environment of the ocean (Sci. Obj. 6) and help infer ejection and freezing conditions from the composition of plume material (Sci. Obj. 8.2) by measuring the soluble ionic species in the melted plume ice grains and also determining average and individual-species redox potentials, salinity, and pH. The Wet Chemistry Lab (WCL) on *Phoenix* was the first of this kind of spaceflight instrument; it successfully operated on the surface of Mars (Hecht *et al.*, 2009; Kounaves *et al.*, 2009, 2010).

Recent developments—for example, the microfluidic Wet Chemistry Lab (mWCL) and Sample Processor for Life on Icy worlds (SPLIce) supported by NASA-COLDTech and the Microfluidic Icy-World Chemistry Analyzer (MICA) supported by the NASA ICEE-2 program—employ microfluidic engineering to decrease the volume of both the instrument and the sample needed (Chinn *et al.*, 2017; Chin *et al.*, 2018; Noell *et al.*, 2019; Radosevich *et al.*, 2019). The fluidics system that integrates the sensor array of the ESA would maximize the synergies with the capabilities and functions of the sample transfer and processing system; some critical measurements, for example, pH and ionic conductivity, are purposefully redundant to increase reliability and provide limited dual-string capability with minimal mass and complexity penalties.

Sci. Obj. 1, 2, and 3 would also be addressed with measurements by a capillary electrophoresis-laser-induced fluorescence microfluidics device. Fluorescent reagents specific to pendant functional groups would be mixed with the sample solution, passed through a capillary electrophoresis system to separate compounds by charge and by size, and finally analyzed via laser-induced fluorescence. This yields information on the concentration (from fluorescence intensity) and compound identity (based on retention time) (*e.g*., Stockton *et al.*, 2009a, 2010, 2011). In this study, we target molecules containing amino and/or carboxylic acid groups, which can be identified with better than nanomolar sensitivity (sub-ppb) (Mathies *et al.*, 2017).

Chirality would be measured via micellar electrokinetic chromatography (Chiesl *et al.*, 2009). These nondestructive techniques rely on concentration rather than mass to achieve high sensitivity through sample accumulation and are, thus, specifically complementary to the SMS and HRMS investigations. Several independent and collaborative efforts are developing these kinds of instruments (Mathies, University of California, Berkeley via NASA ICEE-2 and MatISSE programs; Stockton, Georgia Institute of Technology via NNX15AM98G and NNX16AM82H; Creamer *et al.*, 2017, 2018; Jet Propulsion Laboratory via NASA PICASSO program).

A solid-state nanopore sequencer would address Sci. Obj. 4 by detecting and characterizing linear polyelectrolytes. The liquid sample would be passed through a flow cell with synthetic pores with an applied electric field across the flow cell plane. Polyelectrolyte chains are made up of repeating sets of polymer features, such as the nucleobases A, T, G, and C of a DNA polymer. When a polyelectrolyte passes through the pore, different types of features induce a current change, and the magnitude and direction of that change (positive or negative) can be indicative of that feature.

Thus, the successive changes in current can potentially identify both the single polymer features and their ordered sequence. The powerful science return of this kind of analysis makes this instrument a key component of the Orbilander LDS, despite being currently at low TRL. The development of solid-state synthetic nanopore sequencers includes NASA programs, academic groups, and commercial enterprises (*e.g*., Carr, MIT via PICASSO; McKay, NASA Ames via COLDTech 80NSSC19K1028), which suggests near-term TRL increases. However, a compelling, Flagship-class astrobiological investigation at Enceladus in the next decade, such as any of the concepts described in this article, does not fundamentally require a solid-state nanopore sequencer, should development not proceed as anticipated.

A microscope would search for evidence of cells (Sci. Obj. 5). Given the relative ambiguity of relying on morphology alone for cell identification, measuring a second cell characteristic coincident with promising morphology is key to collecting less ambiguous evidence (Hand *et al.*, 2017; Nadeau *et al.*, 2018). For the Orbilander, we chose autofluorescence as the second characteristic as it does not require viable cells (Bhartia *et al.*, 2010; Hand *et al.*, 2017). More study is needed to elucidate whether autofluorescence would be diagnostic given the expected organic content of the plume or whether/which fluorescent tags should be employed. Fluorescent minerals can be disambiguated from the context of other instruments, such as the context imager (whose filters could be chosen specifically to provide this context) or the composition of plume material determined from the mass spectrometers and ESA.

Alternative cell characteristics could be considered, especially as technical maturity continues to advance. Digital holographic microscopes are capable of distinguishing biological from Brownian motion (Nadeau *et al.*, 2016; Serabyn *et al.*, 2016; Bedrossian *et al.*, 2017). Atomic force microscopes (AFM) can probe the biomechanical properties of cell candidates (Dorobantu *et al.*, 2012) and have been flown for non-astrobiology science objectives (Barth *et al.*, 2001). The Orbilander microscope is modeled after the requirements described by the Europa Lander Science Definition Team (ELSDT) without the AFM component (Hand *et al.*, 2017) and is based on the *Phoenix* MECA microscope (Hecht *et al.*, 2008).

Several groups are funded to develop microscopes specifically for astrobiological (rather than geochemical) investigations, microscopes that utilize multiple excitation wavelengths (including deep-UV) to excite biological autofluorescence and fluorescent stains that target specific structural and functional biomarkers (membrane lipids and proteins) (Quinn *et al.*, 2019) and digital holographic imaging (Kim *et al.*, 2020).

### 3.2. Remote sensing and reconnaissance suite

Here, we describe a model payload for the remote sensing and reconnaissance suite by analogy with similar instruments that have flown or are in development. The scientific drivers described are limited to those listed in Table 1, which is a non-exhaustive list of science objectives that could be accomplished with this set of instruments. For example, in addition to the remote sensing science objectives, these instruments are also part of the notional payload to identify a suitable landing site for the Orbilander (MacKenzie *et al.*, 2020).

A Narrow-Angle Camera (NAC) would provide the sub-meter resolution imaging required to characterize surface topography (Sci. Obj. 8.1), surface expression of the vents (Sci. Obj. 8.3), and to identify a safe landing site. The concept NAC is modeled on the *New Horizons* LORRI camera (Cheng *et al.*, 2009) that has sufficient resolution in a compact athermal design. However, to avoid smearing of high-resolution images at the spacecraft's orbital speed near periapse at Enceladus, modifications would be necessary to accommodate the required short exposure times of ∼1 ms and low light intensity.

A Wide-Angle Camera (WAC) would provide the broad coverage of the SPT at coarser resolutions to allow the science and engineering teams to identify candidate landing site targets for the NAC observations. In the architectures described in this study, either of the two navigation cameras co-boresighted with the NAC would serve as the WAC, providing redundancy for this mission-critical component and ensuring the accomplishment of landing site reconnaissance in a timely manner. For landed architectures, the most demanding WAC requirements would come from navigation rather than science. The Orbilander WAC is modeled after ECAM-M50 from Malin Space Science Systems, a monochrome detector with electronics and optics similar to instruments that have flown on several previous missions.

A Thermal Emission Spectrometer or thermal mapper (TES) would enable measurements of surface thermal emission to inform the physical structure at the jet vents (Sci. Obj. 8.3) by mapping surface temperatures and associated heat fluxes, and determining the thermal properties of surface material from temperature variations (*e.g*., Howett *et al.*, 2011). The TES would also ensure the safety of the landing site. The resulting temperature maps must have temperature sensitivity of ∼1 K and instantaneous field of view <2° to image the landing site candidates (5 × 5 km) at least two pixels across. In this study, the TES is modeled on MERTIS on *BepiColombo*; other examples of this kind of instrument include OTES on *OSIRIS-REx* (Christensen *et al.*, 2018), TES on *Mars Global Surveyor* (Christensen *et al.*, 2001), and E-THEMIS on *Europa Clipper*.

To map the surface topography for Sci. Obj. 8.1 and 8.3 and to identify a safe landing site, the concept payload includes a laser altimeter modeled after OLA, the laser altimeter on *OSIRIS-REx*. The requirements for the laser altimeter are driven both by science (10 cm vertical resolution, sub-m spatial resolution) and by hazard avoidance during landing. In addition to OLA at Bennu, laser altimeters have flown to Mars (Zuber *et al.*, 1992), Mercury (Cavanaugh *et al.*, 2007), and the Moon (Smith *et al.*, 2010), and they are planned to fly to the jovian moons (Kimura *et al.*, 2019).

A radar sounder would address Sci. Obj. 8.1 and 8.3 from orbit with 10 m vertical resolution, driven by the anticipated physical structures of the vent and crust. Notably, Enceladus’ SPT is presumed to be thinner (5 km at the SPT) (Čadek *et al.*, 2016; Thomas *et al.*, 2016), potentially in a melting regime (*i.e*., crustal thinning at the poles), and more uniform in depth when compared with Europa where complex thermal and chemical horizons are expected to be distributed through the ice shell (*e.g*., Soderlund *et al.*, 2020).

Because of the number of unknowns surrounding how and where fissures and vents operate in the subsurface of Enceladus as well as their structure, we selected a 60 MHz radar system modeled after the very-high-frequency element of *Europa Clipper'*s REASON (Moussessian *et al.*, 2015). This radar system can also be used to detect shallow brines, characterize the surface roughness statistically, and determine the dielectric constant as a proxy for near-surface porosity and load-bearing capability to assist with landing site selection (Grima *et al.*, 2014, 2016).

Since the surface return additionally provides altimetric measurements (*e.g*., Steinbrügge *et al.*, 2018), the radar sounder would further add robustness to the laser altimeter. The achievement of the vertical resolution requires 15 MHz bandwidth, giving 25% relative bandwidth with a 60 MHz carrier frequency. A log-periodic dipole array 2 m long and 1.42 m at the longest crossbeam can satisfy these requirements. The 2 m boom could be deployed post-EOI, though calibration during Enceladus flybys necessary to get into Enceladus orbit could enhance the science return. Further study into the specific requirements for and implementation of a sounding investigation at Enceladus is particularly warranted given the uniqueness of the crust (thinner, different thermal gradients, potentially high porosity layers, *etc.*) relative to that of the Galilean satellites that have been studied for *JUICE* and *Europa Clipper*.

### 3.3. *In Situ* Suite

Selecting the target site for the active sampler requires a context imager that is capable of resolving the 1.5 m in front of the instrument panel at 500 μm per pixel to discern the largest grains. At least 50% overlap is necessary for stereo coverage. A simple white-light LED lamp would facilitate imaging in the low light conditions expected during the landed phase of the mission. The concept context imager is modeled after ELSSIE, a context imager designed for the Europa Lander (PI Murchie, JHU APL), which is also capable of conducting a spectroscopic investigation to characterize sample provenance.

The latter is not a driving requirement for the architectures investigated here, as the surface materials we target are plume fallout materials for which we would already have some understanding from the compositional analyses conducted in the orbital phase. Examples of other context imagers include the Stereo Surface Imager on Phoenix (Lemmon *et al.*, 2008) and C-LIFE (PI Byrne, University of Arizona).

A seismometer would address Sci. Obj. 8.1 and 8.3. Notably, these are the same objectives addressed by the radar sounder in a highly complementary manner. The seismometer would be sensitive to structural transitions at much greater depths than the radar sounder (especially since we have chosen a higher frequency carrier to resolve the near-surface crust at higher vertical resolution) and to the dynamics of the interior in real time. Tidally driven seismic events on Enceladus of the same order of magnitude as lunar seismic events should occur at least twice an Earth month (Hurford *et al.*, 2020); other possible sources of activity include ice shell fracturing, ocean currents, and geyser activity (Stähler *et al.*, 2018; Vance *et al.*, 2018). Monitoring the timing and location of these events could reveal further details of the interior structure of Enceladus.

Noise estimates for seismic activity at Europa suggest that high-frequency geophones may be sufficient seismic probes there (Panning *et al.*, 2018); a dedicated study of the conditions at Enceladus would be needed to inform a seismometer-geophone trade. For example, tidally induced events at Enceladus are predicted to be 3 orders of magnitude lower than at Europa (Hurford *et al.*, 2020); however, since Enceladus is a smaller body, each event is subject to less geometric spreading. In the absence of such studies, we use the requirements outlined in Vance *et al.* (2018) for the seismic investigations of the architectures explored here.

A short-period seismometer capable of monitoring frequencies 0.1–10 Hz, such as the SEIS-SP on InSight (Lognonné *et al.*, 2019), served as our model implementation. The development of seismic packages specifically for Ocean World deployment is currently underway via PICASSO (PI Yee, JPL), MatISSE (PI Chui, JPL), ICEE2 (PI Bailey, University of Arizona; PI Panning, JPL), and COLDTech (PI Yu, Arizona State University) programs.

## 4. Sample Requirements and SS

Plume material must be acquired for the LDS analyses—how much depends on both the instrument functionality and the expected organic abundance and potential biomass in the ocean. The former is well known for the high-TRL instruments selected for the model payload. The latter, however, is highly uncertain as it depends on assumptions of the style and extent of water–rock reactions at the ocean–sediment interface, as well as on assumptions regarding the availability of energy sources for life (Cable *et al.*, 2020; Ray *et al.*, 2021).

Similar to the strategy of the ELSDT study report (Hand *et al.*, 2017), we modeled the organic abundance and potential biomass of Enceladus’ ocean based on analogies to terrestrial values. Unlike the ELSDT report, however, our estimates are also anchored in observations (by the *Cassini* spacecraft) of the plume's organic content. One of the key drivers of a surface phase for the Orbilander mission was the increased access to plume material: It is possible to sample a larger volume of falling or fallen plume material from the surface. Increased sample access significantly increases the expected signal-to-noise ratio and would help retire some of the risk associated with uncertainties in the assumed organic content and biomass of ocean materials. We present our choices in Table 6 and describe our rationale in this section.

### 4.1. Organic abundance and potential biomass assumptions

We modeled the expected total organic carbon (TOC) of Enceladus’ ocean as 30 μ*M* (1 *M* = 1 molar = 1 mol per liter of H_2_O), a value similar to that of Earth's circumpolar deep water and of seawater from Central North Pacific and Sargasso Sea (Kaiser and Benner, 2008), and on par with the ELSDT model for Europa's subsurface ocean (41 μ*M*). Unlike at Europa, our model can be compared with *in situ* data from *Cassini'*s Cosmic Dust Analyzer.

In Saturn's E-ring, 8% of the organic-rich (“Type 2”) grains have an organic concentration of ≥1 m*M* (Khawaja *et al.*, 2019). Type 2 grains should be more abundant at lower altitudes than in the E-ring, where they represent 30% of the plume content. Thus, *Cassini* data suggest that the overall concentration of organic material in the plume is higher than 0.08 × 1 m*M* × 0.3 = 24 μ*M*, a lower bound in line with our terrestrial model, noting that the concentration of organics in the plume material does not necessarily map 1:1 to the expected abundances in the subsurface ocean.

Changes in the overall concentration and relative abundances of organic compounds are likely to result from the mechanics of ejection. Proposed possibilities include bubble scrubbing (Porco *et al.*, 2017), bubble bursting at the liquid–vacuum interface (Porco *et al.*, 2017; Postberg *et al.*, 2018a), condensation and/or adsorption of volatile organic compounds onto ice grains within the vents (Postberg *et al.*, 2009; Bouquet *et al.*, 2019; Khawaja *et al.*, 2019), or concentration of bioessential elements at the ice–ocean interface due to ice crust recycling (Teolis *et al.*, 2017; Cable *et al.*, 2021; Ray *et al.*, 2021).

We then set the expected abundance of amino acids to 150 n*M* by using the 1:200 ratio of protein to organic content in Earth's oceans. This terrestrial ratio comes from considering the volume of protein per cell (25 fg protein/cell) (Zubkov *et al.*, 1999), the concentration of cells in ocean water (10^5^ cells/g water) (Whitman *et al.*, 1998), and the concentration of total organic matter in the oceans (0.5 ppm by mass) (Thurman, 1985). This is less conservative than the ELSDT's 1:400 ratio, but less optimistic than Steel *et al.* (2017) whose model predicts tens of μ*M* of amino acids. For lipids, we again employ a terrestrial ratio of lipids to amino acids in cells, 1:5.

Notably, these assumptions do not take into account the relative abundance of free or bound (in polymers, within cells, *etc.*) organic material, the ratio of which is unknown for Enceladus’ ocean. Should abiotic processes dominate, for example, free organics may be at higher abundance than found in Earth's oceans. This uncertainty motivates the choice of instrument types that have low limits of detection (LODs) and to accommodate their respective sample processing needs (Section 4.3).

The concentration of free DNA in Earth's oceans is ∼100 ng/L (Collins *et al.*, 2018). Unlike smaller precursor molecules, free DNA will require biological processes to be present in Enceladus’ ocean. This is the critical unknown, but we derive a useful estimate for an Enceladus value by considering that, as detailed in Cable *et al.* (2020) and discussed later, the lower energy available to a putative Enceladus biosphere leads to a lower biomass density. We, therefore, decrease the terrestrial concentration by two orders of magnitude and assume a concentration of free biopolymer material in Enceladus’ ocean ∼1 ng/L.

Salinity, pH, and Eh were based on *Cassini* results (*e.g*., Postberg *et al.*, 2008, 2009, 2011; Hsu *et al.*, 2015; Waite *et al.*, 2017) and geochemical models of Enceladus’ ocean (Glein *et al.*, 2015, 2020; Sekine *et al.*, 2015) whereas macro- and micro-nutrient abundances (CHNOPS-bearing compounds and elements such as Mg, Ca, and Fe, respectively) in the m*M*-μ*M* range were informed by Cassini results (Waite *et al.*, 2009; Postberg *et al.*, 2011) and by geochemical models of Enceladus’ ocean (Zolotov, 2007).

We assumed that the cell concentration in Enceladus’ plume is ∼10^3^ cells/mL based on model predictions and terrestrial analogues (Table 5). The biomass concentration in plume material might be two to three orders of magnitude higher if plume enrichment processes such as bubble scrubbing are at play (Porco *et al.*, 2017). Our assumption is higher than that of the cell concentrations similarly derived for the surface of Europa, ∼100 cells/mL (Hand *et al.*, 2017), which reflects the difference in the provenance of the Enceladus samples as ejecta from the ocean.

**Table 5.** Considerations of Cell Abundances Used to Inform This Study

Cell concentration (cells/mL)	Location	Description	References
100	Near subsurface ice of Europa	Informed by consideration of Lake Vostok accretion and glacial ice (80–260 cells/mL) (Christner *et al.*, 2006)	Hand *et al.* (2017); see their Table 3.1
10^4^–10^7^	Enceladus plume	Assuming cell concentrations at Enceladus hydrothermal vents are comparable to terrestrial values (∼10^5^) (Brazelton *et al.*, 2006; Perner *et al.*, 2010; Reveillaud *et al.*, 2016) based on a comparison of the ratio of energy fluxes at the sea floor	Porco *et al.* (2017)
80–4250	Ambient Enceladus ocean	Derived from a model of energy flux, hydrothermal H_2_ production consistent with Cassini observations and considering both abiotic and biotic production of amino acids	Steel *et al.* (2017)
8.5 × 10^7^	Enceladus plume		
10^9^	Enceladus vents		
0.6–890	Ambient Enceladus ocean	Derived from the reported energy flux of hydrothermal H_2_ production of the Vance *et al.* (2016) model, assuming methanogenesis	Cable *et al.* (2020)
6 × 10^−6^–0.12	Ambient Enceladus ocean	Derived from the reported energy flux of hydrothermal H_2_ production of the Taubner *et al.* (2018) model, assuming methanogenesis	

**FIG. 2.**
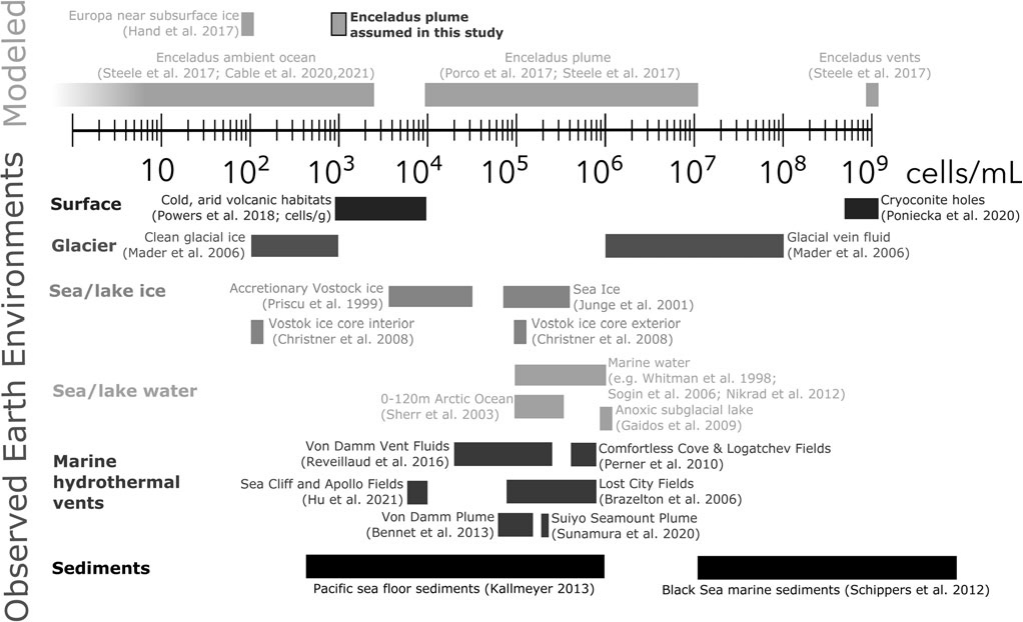
Non-exhaustive examples of terrestrial biomass density compared with model predictions of ocean worlds described in Table 5.

Our assumed cell concentration is, however, two orders of magnitude lower than the biomass density in the Earth's oceans (∼5 × 10^5^ cells/mL) (Whitman *et al.*, 1998). A comparison of the range in biomass density spanned by (a non-exhaustive list of) example environments on Earth to modeled ocean world environments is presented in Fig. 2.

Thus, although our philosophy was to take a more conservative stance than predictions for Enceladus available in the literature, it should be noted that these models are grounded in estimates of the energy available to support a biosphere and are therefore subject to the same level of uncertainty surrounding that energy flux. Because of Cassini's *in situ* investigations, we have more observational constraints—such as tidal energy dissipation (Choblet *et al.*, 2017; Lainey *et al*., 2020) and flux of H_2_ observed in the plume (Waite *et al.*, 2017)—than for the oceans of other Ocean Worlds.

However, so many unknowns remain that predictions for energy fluxes vary by four orders of magnitude (Cable *et al.*, 2020). In the mission concepts built from the science objectives presented in this article, we therefore considered the impact of lower-than-assumed cell concentrations. The details differ somewhat between architectures and are described in MacKenzie *et al.* (2021). For the Orbilander architecture, where a single spacecraft first orbits Enceladus then lands, we maximized sample collection—and thus the potential for concentration—by including both passive and active sampling mechanisms.

The active sampling mechanism was assumed to excavate 5 mL per scoop. Thus, a single scoop should provide enough sample for microscopic imaging of a statistically significant number of cell morphologies even at lower-than-assumed cell abundances. Operational margin to accommodate additional sample collection time is another useful tool for ensuring robustness to the uncertainties in assumptions of biomass.

### 4.2. Instrument requirements

The volume of sample a given architecture must collect will be a function of the chosen payload. For this concept, we used values for the LOD and volume necessary to run a measurement for each instrument type from published values for analogous instruments that have flown or from experts’ best estimates for analogous instruments in development (Table 6). The sample requirements for each instrument of the LDS (except the nanopore) were then derived by convolving the earlier assumptions of ocean abundance with instrument requirements (for signal-to-noise ratio of 3, and the volume necessary to run the measurement):

**Table 6.** Sample Requirements for Each Measurement and Instrument of the Life Detection Suite Derived for This Study

Payload element	Target measurement	Expected ocean abundance		Instrument requirements						No. of independent analyses	Total plume material	
				Instrument LOD^a^		Measurement volume		Sample volume^b^				
HRMS	TOC (Sci. Obj. 1.A-C; 6.1B; 6.2; 6.3; 6.4; 7D; 8.2A)	3E-11	mol/μL	5E-15	mol/μL	1	μL	0.0005	μL	5	0.0025	μL
SMS	Amino acids (2.A-B)	1.5E-13	mol/μL	2E-11	mol	1		400		5	2000	
	Lipids (3.A)	3E-14	mol/μL	1E-12	mol	1		100			500	
μCE-LIF^c^	Amino acids (2.A-B)	1.5E-13	mol/μL	3E-16	mol/μL	6		0.04		5	0.2	
	Lipids (3.A)	3E−14	mol/μL	3E−16	mol/μL	6		0.2			1	
ESA	6.4C Macro-nutrients (oxidants and reductants)	1E-8	mol/μL	1.00E-10	mol/μL	50		1.5		5	375^d^	
	6.4A Micro-nutrients (inorganic ions)	2E-10	mol/μL	1.00E-10	mol/μL			75				
	6.1A pH	8–12		2–14				n/a				
	6.3 Salinity	0.5–3	%	0.01–30	%			15				
	6.4B Eh	−1.0 to 1.0	V	−1.0 to 1.0	V			n/a				
Microscope	Cells (Sci. Obj. 4)	1	cells/μL	1	cells	1		1		5	5	
Contingency total								0.6	mL		2.96	mL
Nanopore	Polyelectrolyte (Sci. Obj. 5)	1E-12	g/μL	1E-15	g/μL	10		10^e^		3	30	
Full total								10.6			33.0	

^a^ Based on performance reported in the peer-reviewed literature (see text for references).

^b^ Calculated as the volume needed to detect a concentration 3 × LOD, that is, sample volume = 3 × LOD/[expected ocean abundance] × [measurement volume], except for the nanopore volume. If the sample volume exceeds the measurement volume, the sample needs to be concentrated (*e.g*., by vaporizing and venting excess water) before analysis.

^c^ Differences from Orbilander report reflect updated understanding of instrument capabilities.

^d^ After multiplication of only the highest sample volume (75 μL) as all ESA measurements of a given sample are made on the same aliquot.

^e^ Calculated as 1 × LOD due to high sample demand and lower confidence in instrument capabilities given by low TRL.

LOD = limit of detection; TOC = total organic carbon; TRL = Technology Readiness Level.


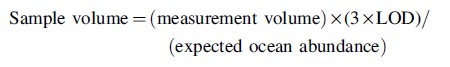


Notably, at this level of study we did not fold in the volume of liquid necessary to flow the collected sample through the sample preparation and transfer system. For example, front-end sample handling systems currently in development require volumes larger than some instruments require, on the order of 20–50 μL (*e.g*., Chinn *et al.*, 2017; Chin *et al.*, 2018). Thus, the total sample volume requirements would likely change with a more detailed model of the sample handling and preparation—especially considering whether the concentration before delivery to each instrument is possible or required—which was beyond the scope of this concept study.

Such an effort should take advantage of the lessons learned from efforts for designing an SS for a Europa Lander, while designing specifically for the unique challenges and advantages of the Enceladus surface (*e.g*., Badescu *et al.*, 2019; Backes *et al.*, 2020; Choukroun *et al.*, 2020, 2021).

Since solid-state nanopore systems are at a relatively low TRL at the time of this study, we describe here in detail how the requirements for this instrument were defined. We assumed a solid-state nanopore system that can withstand a long spaceflight duration with at least four wells delivering at least four synthetic nanopores (and thus the capability for four independent analyses, with this number being limited by data storage and data transfer rates; see appendix A of MacKenzie *et al.*, 2021).

Sample preparation steps necessary for biopolymer detection and sequencing were assumed to be part of the instrument itself but could be levied on the sample preparation system.

State-of-the-art nanopore sequencing systems can detect 10^−3^ pg biopolymer (equivalent to 10^3^ reads of average length 10^3^ bases, or 10^6^ bases) in a sample that contains 1 ng biopolymer per mL (Carr *et al.*, 2017). The expected concentration of biopolymer in Enceladus’ ocean, extrapolated from comparisons with the Earth's oceans, is 1 pg/mL. To obtain 1 ng biopolymer per mL, the original sample must be concentrated by a factor of 1000. Since the sample volume required to perform a nanopore analysis is 10 μL, the minimum original sample volume for an exotic biopolymer detection at Enceladus is 10 mL.

### 4.3. SS requirements

There are five key steps to ensure successful sample delivery to the LDS: (1) collection, (2) delivery to the central processing unit, (3) processing, (4) delivery to instruments, and (5) flushing the system clean for the next analysis. Thus, an SS is also a required element of the payload.

#### 4.3.1. Collection

We modeled the passive ice particle collector as a 1 m^2^ funnel (Adams *et al.*, 2018), which would be employed during plume flythroughs and on the surface. The funnel opening would be protected by a reclosable cover during cruise and pre-landing descent. Collecting ice particles at the low relative velocity of the orbit and at near-zero relative velocity when landed eliminates impact-induced changes to the sample: Particles in the plume travel at velocities 100–200 m/s (Guzman *et al.*, 2019), whereas Orbilander's translational velocity is up to 200 m/s.

The modeled funnel has been tested for collection at up to 2 km/s. Alternative approaches for collection may be suitable, such as an impact capture plate (Mathies *et al.*, 2017) that has been demonstrated at hypervelocities to efficiently capture ice particles and their entrained organics with minimal decomposition, with data suggesting improved capture efficiency at the lower relative velocities of Enceladus orbit (New *et al.*, 2020).

A gas inlet is also included in the concept design to allow vapor to pass into the HRMS during plume flythroughs. A cover would prevent contamination during cruise. Descent contamination of the inlet is possible but not of concern, as on the surface the prime sample for the HRMS would be vaporized ice grains supplied by the SS. Active sample collection would only be conducted on the surface and is modeled as a scoop capable of retrieving 5 cm^3^ of surface ice.

Optimization of the active collector (*e.g*., scoop, rasp, drill, pneumatic transfer) for the cold, low-gravity Enceladus environment was beyond the scope of this study but should be addressed in the next phase of the study.

#### 4.3.2. Delivery to interior

The funnel and scoop would each have separate cups for receiving samples. These receiving mechanisms must be kept cold enough to minimize sample modification before analysis. When ready for analysis, the cups would be sealed and brought into the interior. Alternative arrangements that minimize transfer mechanisms may be considered, such as the CADMES concept under development for Europa Lander (*e.g*., Malespin *et al.*, 2020). Some means of estimating the amount of sample acquired would also be useful, informing distribution to the instruments downstream.

#### 4.3.3. Processing and delivery to instruments

Sample preparation is an essential and often unappreciated requirement for sensitive life detection experiments (Willis *et al.*, 2015), particularly in the context of ocean worlds where organic matter could be bound to particles (mineral and organic), exist within protective layers such as spores or cell walls, or be polymerized (*e.g*., Toner *et al.*, 2016). For that reason, analytical instruments that target individual molecules (*e.g*., amino acids, lipid hydrocarbons, polyelectrolytes) require sample preparation steps that disaggregate macromolecular carbon and separate molecules from minerals.

The latter case might be less relevant for the case of Enceladus, where mineral particles are expected to be rare in the ocean column. The salts and silica nanoparticles detected by *Cassini* are perhaps the leading exception, though their binding potential is not yet fully understood. At the pH expected for Enceladus’ ocean, the net negative charge of the silica grains may inhibit the adsorption of anions such as carboxylic acids, or very small particle sizes may create high surface free energies that could favor enhanced adsorption.

The concept process for Orbilander begins with heating the sealed cups to melt the ice. The resulting liquid would be transferred through microfluidic tubes to the Sampling Processing Subsystem (SPS). Careful laboratory testing of potential organic matter analogues (chondritic insoluble organic matter, humic acids, methanogen biomass, *etc.*) should be conducted to understand any fractionation effects of melting.

The SPS would either deliver pristine liquid directly to an instrument or conduct preparation steps depending on the needs of the instrument (*e.g*., Chinn *et al.*, 2017; Chin *et al.*, 2018; Kehl *et al.*, 2019; Radosevich *et al.*, 2019). Derivatization reactions, for example, are key for the amino acid and lipid hydrocarbon analyses conducted with instruments such as the microcapillary electrophoresis with laser-induced fluorescence (μCE-LIF) and the SMS.

These reactions can help break down bound organic matter (*e.g*., Mora *et al.*, 2020), making both free and bound organics available for analysis. For the SMS, chemical derivatization or thermochemolysis (heat-assisted derivatization) has been used on Earth and Mars to make both bound and free organics available for analysis; He *et al.* (2020) provide a recent review. Preparation steps for the μCE-LIF could include application of aqueous buffers (used by Stockton *et al.*, 2009a, *e.g*., on briny artic samples) and/or soluble reagents (such as Pacific Blue) (Kaiser *et al.*, 2013). Reagents can even be applied to solubilize hydrophilic compounds such as PAHs by using soluble cyclodextrans (Stockton *et al.*, 2009b). Micellar chromatography has also been used (Chiesl *et al.*, 2009) to preferentially solubilize amine-containing molecules.

Other preparation steps may include filtering, division, extraction, tagging, concentration, de-salting, de-bubbling, and/or characterization of properties such as salinity and pH. Concentration and extraction will be especially important for the polyelectrolyte search with the nanopore; efforts to tailor well-established terrestrial laboratory techniques such as mechanical lysis are already underway for Mars (Mojarro *et al.*, 2017) and Ocean World exploration (Craft *et al.*, 2020).

Liquids would be delivered to the ESA, μCE-LIF, microscope, and nanopore via microfluidic capillary tubes. The SPS would provide vapor to the SMS and HRMS by vaporizing the liquid sample. The model SMS contains a hermetically sealed supply of ultra-pure water, inert gases, and dry reagents (including calibrants and standards) for sample preparation.

Housing common preparation techniques in one unit achieves an advantageous minimization of mass, power, and volume resources (*e.g*., efficient dual-string implementation of certain key components such as pumps and sensors). Successful implementation requires an early, coordinated effort between instrument teams, which would be facilitated by continued development of microfluidic preparation and delivery systems (*e.g*., Zhong, JPL via PICASSO; Short, SR via PICASSO; Malespin, NASA GSFC via ICEE2; Ricco, NASA Ames via PICASSO, COLDTech; Mathies, Berkeley, via COLDTech and ICEE2; Bourouiba, MIT via PICASSO; Glein, SwRI via ICEE2). We modeled enough supplies for more than twice the required samples.

Although sample handling for flight missions is an active area of innovation, more investment might be needed to transfer sample preparation steps commonly used in the laboratory into flight instrumentation. Based on the current state of the art (*e.g*., Chinn *et al.*, 2017; Chin *et al.*, 2018; Radosevich *et al.*, 2019; Mora *et al.*, 2020) and assuming continued programmatic support, there are reasons to feel optimistic that adequate sample preparation technology will be matured within the timeframe of the Orbilander mission.

#### 4.3.4. Flushing

A reservoir of sterile, contaminant-free solution would flush the SS in between analyses.

### 4.4. Sampling operations

**FIG. 3.**
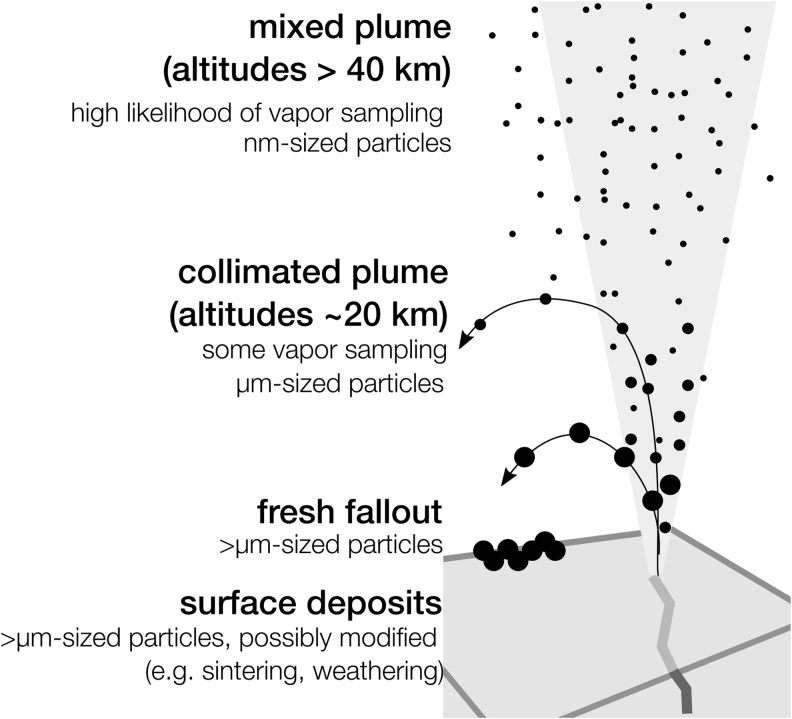
Particle size distribution, and therefore organic matter abundance, differs with location in the plume, motivating the capability of sampling from orbit and on the surface.

The Enceladus plume can be considered as four different populations of material (Fig. 3). At altitudes ≳40 km, the individual jets mix, creating a more favorable environment for vapor sampling but particle sizes are on the order of nanometers. Below 40 km, the collimated jets contain larger, micron-sized particles (Guzman *et al.*, 2019). On the surface, fresh fallout of larger particles (too heavy to achieve escape velocity) can be intercepted with a passive collection mechanism.

Finally, surface deposits are also available, offering the opportunity to rapidly collect large sample volumes with an active sample collector. However, these deposits may have experienced some modification from weathering (Bergantini *et al.*, 2014) or sintering (Choukroun *et al.*, 2020). Further work is needed to determine the extent of alteration of surface materials by processes such as this, and how these relate to the rate at which these materials are covered up by deposition of fresh plume material. There are several solutions for avoiding surface contamination from thruster firing during descent, should it be of concern (MacKenzie *et al.*, 2021).

In Enceladus’ orbit, Orbilander velocity in the ram direction would peak at ∼200 m/s, about the escape velocity of jet particles. Plume flythroughs at these velocities, thus, allow collection with little modification to the sample. Using the model of Guzman *et al.* (2019), we assume that the flux of particulates in these reservoirs is 1.6 μL/(m^2^·orbit). This is conservatively at the lower end of published flux values [Porco *et al.*, 2017, *e.g*., estimate 0.5–6 μL/(m^2^·orbit)]. During each orbit, particulates would be accumulated until a desired volume is reached.

The sample collection system must, therefore, be able to determine how much sample is acquired. The Orbilander concept SS includes this capability. Alternative solutions include microscopic imaging of the collector (if the geometry allows) or of a witness plate. Other solutions include using optical and/or AFM such as those employed by the Mars *Phoenix* lander and *Rosetta* spacecraft, tailored to the expected size and number density of collected particles (Bentley *et al.*, 2016).

Alternatively, one could measure the effect of a changing mass of the collection surfaces on the frequency of a quartz crystal microbalance. This technique is commonplace in monitoring chemical contamination (*e.g*., deposition of organic compounds outgassed from tapes or glues) during spacecraft assembly or even in flight (Dirri, 2018). If the collected mass is significant (*e.g*., landed collection), its effect on the collector or spacecraft inertia could be monitored. This was the designed approach for the *OSIRIS-REx* asteroid sample return mission's goal to collect at least 60 g (Lauretta *et al.*, 2017).

On the surface, the rate of passive collection depends on the distance from jet vents. Predicted fallout rates vary across the surface by orders of magnitude, reaching up to 1 mm/year (Southworth *et al.*, 2019). In this study, we assumed that the spacecraft lands in an area where fallout is at least 0.1 mm/year. The same passive collection mechanism used in orbit would be employed on the surface. The reclosable cover would prevent contamination during landing.

For the purposes of this study, we modeled the active sample collector as a scoop capable of excavating 5 cc of regolith as the active sampling collector, similar to the strategy of the ELSDT. The specific mechanism for excavating and retrieving samples in the cold, low-gravity environment of Enceladus’ surface warrants dedicated study. Rasps or drills (Badescu *et al.*, 2019), perhaps combined with pneumatic transfer systems (Zacny *et al.*, 2019), may prove better suited.

## 5. Conclusions and Recommendations

A return to Enceladus to search for evidence of life is not only well motivated by *Cassini* data but readily achievable with today's technology. As part of the mission concept study of Flagship-class architectures for the 2023–2032 Planetary Science and Astrobiology Decadal Survey, we identified a set of science goals and objectives to answer the questions of whether Enceladus is or has been inhabited and to what extent the subsurface ocean conditions are suitable for supporting life. By targeting biosignatures that span a variety of characteristics of life with instrumentation of overlapping capability, our set of five life detection objectives represents a complementary and orthogonal approach.

The selection of objectives for quantifying Enceladus’ habitability was, in part, driven by the choice to study orbital and landed architectures. Notably, these are not the only conceivable science objectives possible at Enceladus or payload worth flying. Rather, the science traceability matrix shown here is a representation of the scope and possible payload. With continued funding for instrument development and maturation (*e.g*., programs such as MatISSE, PICASSO, SESAME, ICEE, COLDTech), new approaches will emerge. For example, instrumentation capable of measuring compound-specific isotopic abundances would strengthen both the amino acid and lipid characterization objectives, making a life detection claim more robust. However, this study demonstrates that a compelling and robust search for an evidence-of-life mission at Enceladus is possible in the next decade with readily available and in-development instrumentation.

## Abbreviations Used

μCE-LIF: microcapillary electrophoresis with laser-induced fluorescence
a.a.: amino acid
ACE: APL Concurrent Engineering
AFM: atomic force microscopes
CH_4_: methane
CO_2_: carbon dioxide
ELSDT: Europa Lander Science Definition Team
ESA: electrochemical sensor array
GCMS: gas chromatograph mass spectrometer
H_2_: molecular hydrogen
HRMS: high-resolution mass spectrometer
LC-MS: liquid chromatography–mass spectrometry
LDS: Life Detection Suite
LOD: limit of detection
MA: Molecular Assembly
MS/MS: tandem mass spectrometry
NAC: narrow-angle camera
OLA: laser altimeter on *OSIRIS-REx*
SMS: separation mass spectrometer
SPS: Sampling Processing Subsystem
SPT: South Polar Terrain
SS: Sampling System
TES: thermal emission spectrometer or thermal mapper
TOC: total organic carbon
TOF: time-of-flight
TRL: Technology Readiness Level
UV: ultraviolet
WAC: Wide-Angle Camera

## Notes

^*^
House of Representatives 2016 Commerce, Justice, Science, and Related Agencies Appropriations, https://www.congress.gov/congressional-report/114th-congress/house-report/130/1

^*^

https://cosparhq.cnes.fr/assets/uploads/2020/07/PPPolicyJune-2020_Final_Web.pdf

## Notes

Associate Editor: Jack Mustard
